# Access to housing subsidies, housing status, drug use and HIV risk among low-income U.S. urban residents

**DOI:** 10.1186/1747-597X-6-31

**Published:** 2011-11-23

**Authors:** Julia Dickson-Gomez, Timothy McAuliffe, Mark Convey, Margaret Weeks, Jill Owczarzak

**Affiliations:** 1 Department of Psychiatry and Behavioral Health, Center for AIDS Intervention Research, Medical College of Wisconsin, 2071 North Summit Avenue, Milwaukee, WI 53202, USA; 2 Institute for Community Researh, 2 Hartford Square West, Suite 100, Harford, CT 06106, USA; 3 Department of Psychiatry and Behavioral Health, Center for AIDS Intervention Research, Medical College of Wisconsin, 2071 North Summit Avenue, Milwaukee, WI 53202, USA

## Abstract

**Background:**

Much research has shown an association between homelessness and unstable housing and HIV risk but most has relied on relatively narrow definitions of housing status that preclude a deeper understanding of this relationship. Fewer studies have examined access to housing subsidies and supportive housing programs among low-income populations with different personal characteristics. This paper explores personal characteristics associated with access to housing subsidies and supportive housing, the relationship between personal characteristics and housing status, and the relationship between housing status and sexual risk behaviors among low-income urban residents.

**Methods:**

Surveys were conducted with 392 low-income residents from Hartford and East Harford, Connecticut through a targeted sampling plan. We measured personal characteristics (income, education, use of crack, heroin, or cocaine in the last 6 months, receipt of welfare benefits, mental illness diagnosis, arrest, criminal conviction, longest prison term served, and self-reported HIV diagnosis); access to housing subsidies or supportive housing programs; current housing status; and sexual risk behaviors. To answer the aims above, we performed univariate analyses using Chi-square or 2-sided ANOVA's. Those with significance levels above (0.10) were included in multivariate analyses. We performed 2 separate multiple regressions to determine the effects of personal characteristics on access to housing subsidies and access to supportive housing respectively. We used multinomial main effects logistic regression to determine the effects of housing status on sexual risk behavior.

**Results:**

Being HIV positive or having a mental illness predicted access to housing subsidies and supportive housing, while having a criminal conviction was not related to access to either housing subsidies or supportive housing. Drug use was associated with poorer housing statuses such as living on the street or in a shelter, or temporarily doubling up with friends, acquaintances or sex partners. Living with friends, acquaintances or sex partners was associated with greater sexual risk than those living on the street or in other stable housing situations.

**Conclusions:**

Results suggest that providing low-income and supportive housing may be an effective structural HIV prevention intervention, but that the availability and accessibility of these programs must be increased.

## Background

Many studies have found an association between homelessness and housing instability and a variety of HIV risk behaviors compared to similar populations with stable housing. Estimates of the prevalence of HIV among the homeless in the United States range from 3% to 19.5%. In addition, already vulnerable populations of homeless injection drug users, people of color, women and youth experience significantly higher rates of HIV infection [1].

Homelessness and housing instability have been associated with increased numbers of unprotected sex acts [2-4] and increased rates of sexual exchange [3,5,6]. Homeless persons also suffer disproportionate rates of sexual victimization, increasing their risk of contracting HIV [7-11]. Among injection drug users, the homeless have increased rates of needle sharing [5,12], and drug injection [13] and are more likely to frequent shooting galleries [6,14]. Many of these studies are longitudinal [13,15] and thus, document that homelessness often temporally precedes risk behaviors. Homelessness and housing instability also have been associated with poor adherence to Highly Active Antiretroviral Therapy (HAART) medications and poorer health outcomes, both in observational studies and randomized controlled trials [16,17].

As Weir and colleagues point out, one of the limitations in early research on the association between homelessness/unstable housing and HIV risk was the dependence on dichotomous (i.e. homeless versus housed), or trichotomous (homeless, unstably housed, stably housed) measures of housing status [18]. While definitions vary from study to study, homelessness is typically defined as sleeping or living on the streets, in a car, homeless shelter, abandoned building or other places not meant for sleeping, unstable housing as living in transitional housing, a drug treatment facility, or temporarily with family, friends or strangers, and stable housing as living in one's own home.

These narrow definitions often preclude a deeper understanding of the nature of the relationship between housing and HIV risk [18-20]. For example, the affordability of housing is seldom considered and individuals residing in unaffordable housing may resort to opening up their homes to drug using acquaintances who pay a portion of the rent, utilities, or food expenses, or may exchange sex for money [21]. Whether residents have housing subsidies, therefore, may be an important distinction among those who are housed. Likewise, living with family members may increase housing stability compared to residents who live in their own apartment and must therefore face the challenge of paying rent each month. In recognition of the multiple dimensions related to housing, recent research has used more sophisticated measures to capture housing status. These include, for example, distinctions between those who are living with another person compared to those who live in their own apartments [15], and whether participants reside in a supportive housing program and their perceived need for housing services [18].

Many researchers have suggested that federal housing policies may limit access to housing among low-income inner-city residents in general, and particularly among drug users [22,23]. Since the mid-1970s, the dominant model for U.S. federal housing policy has shifted from large housing projects to tenant based vouchers and certificates [24-27]. However, while the federal government provides rental assistance to approximately 4.6 million low-income renters, more than twice as many who are eligible for the programs based on income (9.7 million) receive no federal housing assistance [28]. The federal "One Strike and You're Out" law (P.L. 104-120, Sec.9), passed in 1996, allows federal housing authorities to consider drug and alcohol abuse and convictions by people and their family members when making decisions to evict them from or deny access to federally subsidized housing, although states may ignore this law.

The criminalization of and punitive response to many behaviors in which drug users engage limit their access to employment opportunities, public assistance and subsidized and unsubsidized housing. Public assistance is often discontinued for those in jail, even if not convicted, because they are unable to report for continuing eligibility [23]. Those with criminal histories often find it difficult to find employment. Even those who receive housing subsidies still must find an apartment to rent and many landlords require criminal background checks and may be unwilling to rent to those with a criminal conviction [23,24,29,30]. Other policies that have impacted drug users' access to housing include the Personal Responsibility and Work Opportunity Reconciliation Act of 1996, in particular the elimination of the SSI Addiction Disability and a ban on receiving welfare benefits for convicted drug offenders [31-33]. Little empirical research has examined how these policies may differently affect subpopulations of low-income residents, such as the mentally ill or substance users.

Research has shown that substance use problems are overrepresented among the homeless and afflict anywhere from 28% to 67% of homeless individuals [34-38] and that substance abuse increases individuals' vulnerability to homelessness [39-41]. Cross-sectional research comparing subgroups of homeless have found that homeless with mental illness diagnoses have more access to a variety of public assistance programs that may provide them income to maintain stable housing than homeless without such diagnoses [32,42]. Similarly, Crane and colleagues [31] found that among low-income, marginally housed, former or active drug users, an HIV or AIDS diagnosis actually resulted in improved quality of life by allowing for increased access to SSI, subsidized housing, food and services. However, how housing assistance is delivered is shaped by state and local policies and programs [43]. Thus, personal characteristics such as substance use, criminal convictions, mental illness and HIV may have different effects on access to housing in different states and cities in the U.S.

In response to the multiple social service and medical needs of homeless populations, supportive housing programs have been proposed as a way of increasing housing access and stability for the chronically homeless, including drug users. Supportive housing is permanent, subsidized housing with services such as case management, outpatient drug treatment, employment training, mental health treatment, medical care and social support. Several such programs are being piloted in the Greater Hartford, Connecticut area. Evaluations have shown that, in general, residents of supportive housing programs were able to maintain their housing and decreased their drug use and HIV risk behaviors [44], and increased housing stability among active drug users [45,46]. Randomized controlled trials of supportive housing to people living with HIV/AIDS have shown lower or undetectable viral loads at 1-year follow-up compared to usual care controls [16]. However, similar to housing voucher programs for the low-income, demand for these services far outmatches available resources. Research is needed to understand better how personal characteristics such as mental illness diagnoses, criminal histories, or substance abuse are associated with access to housing subsidies.

In this paper we examine: 1) the effects of personal characteristics (including drug use, income, having a mental illness or being HIV positive) on access to housing subsidies and supportive housing; 2) the relationship between these personal characteristics on housing status; and 3) the relationship between housing status and sexual risk behaviors. This paper adds to the understanding of the relationship between housing and HIV risk by considering how drug use and other personal factors, including income, welfare benefits, HIV diagnosis, and mental illness, may influence access to limited housing subsidies and supportive housing programs and to low-income housing. In addition, our housing status categories capture more detailed nuances of housing statuses, such as whether or not housing is associated with supportive services and implications of differences in relationships with other household members with whom one is living. These distinctions will aid in further understanding the complex relationship between housing status and HIV risk.

## Methods

### Study sites and procedures

We conducted our study in the cities of Hartford and East Hartford, CT, which are separated by the Connecticut River. Hartford is an urban setting with a population estimated at 124,848 people. Hartford has among the highest densities of people living in poverty in the state, with 30% of the households living below the federal poverty line, and a high proportion of ethnic minorities with 72.7% non-white race and 40.5% Latino [47]. It is also a city that lost 15% of its population in the last decade [47]. Some of this exodus includes low-income, ethnic minority residents who have moved to nearby suburbs, including East Hartford. East Hartford has a population of 49,575 and has mixed housing and mixed-income neighborhoods. Twenty-five percent of East Hartford residents are of non-white race, and 15.2% are Latino. Also, 10.3 percent of its population lives below the federal poverty line. In addition, both Hartford and East Hartford include low-income and supportive housing units [47].

We began the study with formative research designed to lay the groundwork for developing a targeted sampling plan [48] of drug using and non-drug using low income residents from the two study towns using data from the 2000 census (the most recent census data at the beginning of the study), data from town property assessors, town planning departments and other sources, "windshield surveys", and key informant interviews with residents and service providers. Targeted sampling is a method to reach hidden populations by recruiting in areas where the targeted population can be found and to reflect the general demographic characteristics of the population.

Using these data sources we constructed the targeted sampling plan. The plan indicated the expected number of low-income residents by gender and ethnicity in each census block group in the two cities that should be represented in our study. Field staff approached potential participants in venues in different census blocks in order to reach a sample that represented the geographic and ethnic diversity of low-income residents in the two cities. Venues included supermarkets, bus stops, on the street, and homeless shelters and soup kitchens for those census blocks which had them.

We conducted face-to-face surveys with 392 drug using and non-drug using low-income residents to measure access to housing and other welfare and health benefits, housing status and stability, drug use and sex risk at baseline, six months and 12 months. Only baseline data are reported in this paper. Potential participants were approached and given an appointment for eligibility screening. Because of the sensitivity of questions to determine eligibility, participants were asked for their written informed consent before screening. Eligibility criteria included being 21 years or older, residing in Hartford or East Hartford, and being low-income. We used the Department of Housing and Urban Development's definition of low-income, based on 50% or less of the median income for a family of four in the Hartford metropolitan area, adjusted according to household size. Ineligible participants were paid $5 for their time. Eligible participants were given coupons and asked to bring up to three people they know who live in their neighborhood to participate in the survey. Survey responses were entered onto handheld computers. Baseline interviews were conducted between October 2008 and August 2010. Participants were paid $25 for completing the survey. All study procedures were approved by Institutional Review Boards at the Medical College of Wisconsin and the Institute for Community Research.

### Measures

*Personal characteristics *measured included gender, race/ethnicity, monthly income from welfare benefits, monthly income from legal employment, total monthly income, education, reporting a mental illness diagnosis, ever having been arrested, ever having a criminal conviction, having spent time in prison, longest prison term served, and self-reported HIV sero-status. Total monthly income included income from employment, welfare sources, informal "under the table work," and illegal activities including selling drugs or exchanging sex. Education was measured as the highest level of education completed. Mental illness was measured with two items, "Have you ever been diagnosed with a mental illness," and the particular diagnosis. In addition we defined recent heavy drug use as having consumed crack, cocaine or heroin in the last 6 months.

We examined *housing access *by independently looking at participants' access to housing subsidies and access to a supportive housing program. For access to housing subsidies, we asked participants whether they had ever received any information about rental subsidies (vouchers to help pay for rent such as Section 8, Shelter Plus Care, Ryan White or Housing Opportunities for Persons with AIDS, HOPWA). For those who had received information about rental subsidies we asked whether they had ever applied for a rental subsidy and whether they had ever received it. For this paper, we treat access as a dummy variable, with 1 defined as applied to and received a housing subsidy, and 0 having never received information about, having never applied for, or applied but never received a housing subsidy. We then asked the same set of questions as with housing subsidies and constructed a dummy variable with 1 having received supportive housing and 0 as having received no information about supportive housing, never having applied for supportive housing, or having applied by not received supportive housing.

To measure *housing status*, we asked participants "Which of the following best describes your *current *housing situations?" Categories included: (1) on the street/in a car; (2) shelter; (3) doubled up with family; (4) doubled up with sex partner; (5) doubled up with friend or acquaintance; (6) in own apartment or home and receives no housing subsidy; (7) in own apartment or home and receives a housing subsidy; (8) supportive housing program; (9) residential treatment program/half-way house/transitional housing; (10) hotel/YMCA (pays rent weekly or monthly with no lease); (11) Single Room Occupancy (SRO)/boarding house; or (12) multiple places. After looking at the numbers of participants in each housing category along with means and standard deviations of sexual risk behaviors associated these (shown in results), we re-classified housing status into 4 categorical variables including: 1) homeless, defined as living on the street, homeless shelter, hotel, car or other place not meant for sleeping; 2) unstably housed, defined as temporarily living with a friend or sex partner, or in an SRO; 3) stably housed, defined as living in your own apartment or in family members' home or apartment; and 4) living in supportive housing.

*Sexual risk behaviors *included: 1) the number of different partners in the last month with whom a condom was not used; and 2) number of times had sex in exchange for drugs, money or something else of value in the last month for constructing the 4-category housing variable. These outcomes were adapted from the Risk Behavior Assessment used in a number of previous studies with active drug users [49,50]. Because 45% of participants reported no sexual activity in the last month we used having any unprotected sex in last month for multiple logistic regressions.

### Analyses

All analyses were conducted using SAS version 9.1 [51] and all significance levels reported were two sided. Personal characteristics found to be significantly associated with access to housing subsidies or supportive housing in univariate regression or Chi Square analyses (p < .1) were included in two logistic regressions to determine the effects of personal characteristics on access to housing subsidies and supportive housing. We hypothesized that drug users and those with criminal convictions would be less likely to receive housing subsidies. Because supportive housing is designed to overcome barriers to accessing housing faced by persons with substance abuse disorders, HIV diagnoses and mental illnesses, we tested whether each of these predicts access to supportive housing. We hypothesized that having a criminal conviction would not be related to access to supportive housing, but that drug use, HIV and a mental illness would predict access to supportive housing.

To adjust for small cell sizes and limited power in the 12 category housing status variable, we looked at the means and standard deviations of number of partners with whom a condom was not used, and number of sex exchanges among the 12 categories to group housing statuses and construct 4 categorical housing variables. Multinomial main effects logistic regression was used to explore the relationship between personal characteristics and current housing status. Because low-income residents who receive housing subsidies still must apply for free-market rental housing, which often includes criminal background and credit checks, we hypothesized that having lower income, a criminal history and drug use would result in less stable housing.

To determine the relationship between housing status and having any unprotected sex in the last 30 days, we performed a multiple logistic regression. The personal characteristics used in the previous models were included in this analysis, except that income from welfare benefits and employment income was used instead of total monthly income, which included income from illegal activities. It is possible that some income may have been generated through sex exchanges, thus conflating our predictor and outcome variables.

## Results

### Representativeness of sample based on targeted recruitment strategy

Our final sample represented 77% of the Hartford census block groups, and 44% of the East Hartford block groups. It should be noted that 71% of the Hartford block groups had 30% or more of the population living below 1.5 times the federal poverty line according to 2000 census data, while only 21% of census block groups in East Hartford had over 30% of its population living under 1.5 times the federal poverty line. Thirty-seven participants were screened but found ineligible, one person refused to participate, and 14 were screened eligible but did not show up to their initial baseline interview.

### Sample Characteristics

We recruited 392 low-income residents from the two cities, including 242 who had used heroin, crack or cocaine within the last 6 months. Our planned recruitment according to race/ethnicity and gender was 36% African American, 52% Latino, and 12% White or Other for Hartford, and 26% African American, 27% Latino, and 47% White or Other in East Hartford. We expected 30% of our sample in both cities to be women, given our over-sampling of drug users. Women drug users in previous research constitute only 20% of our drug using samples. As seen in Table 1 below, African American's and Latinos were slightly over-represented in our sample, as were women. Nearly half the sample had less than a high school education, half had a self-reported mental illness diagnosis, and almost one-third reported that they were HIV positive. The sample was extremely low income. To compare, income at the federal poverty line for a single person in Hartford County is $902.50/month, while the median income of our sample was $560/month. Though all were eligible for rental subsidies based on their income, less than a third of the sample had received a rental subsidy. Even fewer reported ever having received supportive housing and over a third reported having no current source of stable housing. Two-thirds reported that they had a criminal conviction in their lives. In addition, a majority of participants had used heroin, crack or cocaine in their lifetimes, even among those who had not used any of these substances in the last 6 months.

**Table 1:** Sample Characteristics (Percent (n) unless otherwise specified)

	Hartford % (n)	East Hartford % (n)
Gender		
Male	66 (222)	60 (33)
Female	34 (114)	40 (22)
Tansgendered	.003 (1)	0 (0)
Ethnicity	34 (116)	38 (21)
Black/African American	48 (162)	33 (18)
Latino	18 (59)	29 (16)
White/Other		
Total	45.0 (mean) (8.7 sd)	
Age	45.0 (176)	
Less than high school education	$560.00 (mean)	
Monthly income	($424.60) (median)	
Mental illness diagnosis	49.7 (196)	
Self-reported HIV status	25.8 (101)	
Criminal conviction	64.5 (253)	
Ever received rental subsidy	26.5 (104)	
Ever received supportive housing	19.1 (73)	
Homeless	35.5 (139)	
Ever used crack	81.4 (319)	
Ever sniffed cocaine	84.2 (330)	
Ever sniffed heroine	70.7 (277)	
Ever injected cocaine	46.9 (184)	
Ever injected heroine	48.7 (191)	

### The relationship between drug use, personal characteristics, and access to subsidized or supportive housing

Table 2 shows results from two separate multiple logistic regressions showing the relationship between personal characteristics and access to subsidized and supportive housing. As can be seen, there were no significant relationships between drug use and access to either housing subsidies or supportive housing. Being African American and being female was positively associated with receiving housing subsidies. In addition, having an HIV/AIDS diagnosis was positively associated with access to housing subsidies. Contrary to our expectations that having a criminal conviction would be negatively associated with access to housing subsidies, we found no significant relationship between a history of criminal conviction or longest prison sentence served and access to housing subsidies. Access to supportive housing was predicted by having an HIV/AIDS diagnosis or having a mental illness diagnosis. We confirmed our expectation that access to supportive housing was not associated with criminal records.

**Table 2:** Results of logistic regression analysis of access to housing subsidies and supportive housing

	Access to housing subsidy (n = 346)^b^				Access to supportive housing (n = 337)^c^			
	
Predictor^a^	Odds Ratio	95% Wald Confidence Limits	Wald Chi-Square^d^	Pr > ChiSq	Odds Ratio	95% Wald Confidence Limits	Wald Chi-Square^d^	Pr > ChiSq
Drug Use	0.94	0.55 - 1.60	0.06	.82	0.60	0.33 - 1.10	2.77	.096
Female	2.68	1.52 - 4.72	11.63	.0006	0.74	0.38 - 1.45	0.76	.38
Education	0.85	0.58 - 1.24	0.71	.40	1.16	0.75 - 1.78	0.44	.51
African-American	2.41	1.01 - 5.79	3.89	.049	1.98	0.71 - 5.55	1.69	.19
Latino	1.72	0.72 - 4.08	1.50	.22	1.60	0.56 - 4.56	0.78	.38
Monthly Income ($100s)	1.07	1.01 - 1.14	4.49	.034	1.03	0.96 - 1.11	0.64	.42
Told have HIV/AIDS by doctor	2.00	1.13 - 3.54	5.63	.018	5.15	2.75 - 9.66	26.13	<.0001
Mental Illness	1.34	0.79 - 2.27	1.21	.27	1.84	1.00 - 3.39	3.84	.049
Criminal Conviction	3.43	0.45 - 26.4	1.40	.24	0.21	0.01 - 3.58	1.16	.28
Time Convicted	0.76	0.44 - 1.31	0.99	.32	1.37	0.65 - 2.89	0.69	.41

^a ^Predictors: drug use, female, African-American, Latino, told have HIV/AIDS by doctor, mental illness and criminal conviction are coded Yes = 1 and No = 0; education is coded "Less than high school diploma" = 1, "High school diploma or GED" = 2 and "More than high school diploma" = 3; and time convicted is coded "No time served" = 0, "One to 6 days" = 1, "One week or more but less than a month" = 2, "One month or more but less than one year" = 3 and "One year or longer" = 4).

^b ^Access to housing subsidy (coded yes, n = 92; no, n = 254). Hosmer and Lemeshow Goodness-of-Fit Test Chi-Square = 4.37, df = 8. p = .82.

^c ^Access to supportive housing (coded yes, n = 67; no, n = 270). Hosmer and Lemeshow Goodness-of-Fit Test Chi-Square = 7.01, df = 8. p = .53.

^d ^PROC Logistic (SAS v9.2, SAS Institute)--Logistic regression--was used to fit logistic regression models to data.

### Patterns of risk behavior in the 12-item housing status measure

Table 3 shows the number of persons, as well as the mean and standard deviation of sexual risk behaviors for those in each of the 12 housing categories. As can be seen, "doubling up" is not a monolithic category, with those living with family members having fewer instances of sex exchanges compared to those who lived with sex partners, friends or acquaintances. Rather, those living with family showed rates of sex exchange similar to those living in their own apartments. Table 3 also suggests that prevalence of risk behaviors were similar for those who lived in their own apartment with or without subsidies. Participants in supportive housing programs showed similar levels of risk as those living in their own apartments.

**Table 3:** 12-category housing status variable and rates of sexual risk behaviors (N = 392)

*Current Housing Status*	*Total N (%)*	*30 day # partners no condom* *Mean (s.d)*	*30 day # times exchanged sex* *Mean (s.d.)*
On the street/car	2 (0.5 )	0 (0)	0 (0)
Shelter	79 (20.2)	0.4 (0.7)	0.4 (1.3)
Hotel/YMCA	1 (0.3)	0 (0)	0(0)
			
Double Up Sex Partner	33 (8.4)	0.7 (0.5)	1.6 (6.7)
Double Up Friend/Acquaintance	24 (6.1)	0.3 (0.6)	1.9 (9.2)
Single Room Occupancy	11 (2.8)	0.6 (0.5)	0 (0)
Double Up Family	55 (14)	0.3 (0.6)	0.4 (1.2)
Own Apartment, No Subsidy	48 (12.2)	0.3 (0.5)	0 (0)
			
Own Apartment, Subsidy	79 (20.2)	0.4 (0.6)	0.5 (3.4)
Supportive Housing	54 (13.8)	0.1 (0.4)	0.4 (1.3)
Transitional Housing	6 (1.5)	0.2 (0.4)	0 (0)
Multiple Places	0		

### The relationship between drug use, personal characteristics and housing status

Table 4 presents results from a multinomial main effects logistic regression model in which personal characteristics are used to predict the 4-category housing status. As predicted, drug use was negatively associated with living in own home or apartment, or living with a family member. Contrary to our predictions, it was also negatively associated with living in supportive housing. Having been convicted of a crime was negatively associated only with living with a sex partner or friend or in an SRO. However, the longest prison sentence served was positively associated with living with a friend, sex partner or in an SRO. Having HIV was positively associated with living in own home or apartment or living with a family member, and living in supportive housing. Mental illness, however, did not significantly predict any housing status, including living in supportive housing. Income was positively associated with living with a sex partner, friend or an SRO, or living in own apartment or with a family member. Being African American, Latino, and female was associated with doubling up with a sex partner or friend or living in an SRO, or living in own apartment or with family members. Gender and ethnicity did not predict living in supportive housing.

**Table 4:** Results of multinomial main effects logistic regression model showing the influence of demographic factors on housing status

Parameter^a^	Housing category^b^	Coefficient Estimate	DF	Wald ChiSq^c^	Pr > ChiSq	Level comparison	Odds Ratio	Lower 95%CI	Upper 95%CI
									
Intercept^b^									
With sex partner/friend or SRO	2	-0.368	1	0.11	0.739				
With family or in own home/apt	3	1.821	1	3.97	0.046				
In supportive housing program	4	1.274	1	1.24	0.266				
									
Female			3	11.25	0.011				
	2	0.714	1	8.85	0.003	Female vs Male	4.17	1.63	10.68
	3	0.465	1	4.86	0.028		2.54	1.11	5.80
	4	0.121	1	0.22	0.643		1.27	0.46	3.54
									
Black			3	12.99	0.005				
	2	0.596	1	4.89	0.027	Black vs No	3.29	1.15	9.45
	3	0.852	1	12.46	0.000		5.50	2.13	14.15
	4	0.377	1	1.79	0.181		2.13	0.71	6.41
									
Latino			3	25.68	<.0001				
	2	0.708	1	6.57	0.010	Latino vs No	4.12	1.40	12.15
	3	1.219	1	24.85	<.0001		11.46	4.39	29.88
	4	0.428	1	2.01	0.157		2.35	0.72	7.70
									
Education (highest completed)^d^			3	0.29	0.962				
	2	0.110	1	0.14	0.709		1.12	0.63	1.99
	3	-0.019	1	0.01	0.940		0.98	0.60	1.61
	4	0.001	1	0.00	0.997		1.00	0.54	1.85
									
Income ($100s)			3	18.79	0.000				
	2	0.136	1	6.60	0.010		1.15	1.03	1.27
	3	0.182	1	14.56	0.000		1.20	1.09	1.32
	4	0.025	1	0.14	0.708		1.03	0.90	1.17
									
Diagnosis of mental illness			3	4.56	0.207				
	2	-0.363	1	3.19	0.074	Diagnosis vs No	0.48	0.22	1.07
	3	-0.146	1	0.75	0.388		0.75	0.39	1.45
	4	0.052	1	0.06	0.807		1.11	0.48	2.58
									
Told by doctor have HIV/AIDS			3	18.61	0.000				
	2	0.373	1	1.49	0.223	HIV/AIDS vs No	2.11	0.64	7.00
	3	0.699	1	6.90	0.009		4.05	1.43	11.48
	4	1.158	1	15.30	<.0001		10.13	3.17	32.31
									
Ever convicted of crime			3	1.46	0.691				
	2	-0.069	1	0.01	0.937	Convicted vs No	0.87	0.03	25.95
	3	0.553	1	0.61	0.437		3.02	0.19	49.05
	4	0.768	1	0.74	0.390		4.65	0.14	154.43
									
Longest sentence in prison^d^			3	2.35	0.503				
	2	0.101	1	0.05	0.826		1.11	0.45	2.73
	3	-0.372	1	0.96	0.327		0.69	0.33	1.45
	4	-0.435	1	0.83	0.363		0.65	0.25	1.65
									
Recent drug user			3	15.82	0.001				
	2	-0.354	1	2.68	0.102	Drug user vs No	0.49	0.21	1.15
	3	-0.376	1	3.94	0.047		0.47	0.23	0.99
	4	-0.892	1	15.53	<.0001		0.17	0.07	0.41

^a ^Reference category: Female, Black, Latino, Mental illness, Told have HIV/AIDS, Convicted of crime and Recent drug user (no = 0);

^b ^Housing status: On street, in shelter or in hotel/YMCA (reference = 1); Double-up with sex partner or acquaintance or rent single room (2); Double-up with family or living in own apartment/home (3); and In supportive housing program (4).

^c ^Tests of significance for housing status category, individually (DF = 1) and simultaneously (DF = 3), compared with the reference category (On street, in shelter or in hotel).

Education: Less than HS diploma (1), HS diploma or GED (2), More than HS (3); Longest sentence served in prison: None (0), One to six days (1), One week or more but less than a month (2), One month or more but less than a year (3), One year or more (4).

### The relationship between housing status and sexual HIV risk

Table 5 shows the results of a logistic regression testing the effects of personal characteristics and housing status on any sex without a condom in the last 30 days. Those who doubled up with a friend, sex partner or acquaintance were more likely to have had unprotected sex compared to those living on the street, in a homeless shelter or at the YMCA. Having HIV was the only other predictor of sexual risk behavior in this model. Contrary to expectations, having HIV was positively associated with unprotected sex.

**Table 5:** Results of multiple logistic regression analysis of sexual risk behavior in the last 30 days

	Any sex without a condom (117 of 391)		
	
Predictor ^a^	Odds Ratio (95%CI)^b, c^	t	Significance
Female	1.08 (.65 - 1.80)	0.31	.75
Black	1.19 (.60 - 2.36)	0.49	.62
Latino	1.05 (.54 - 2.04)	0.15	.88
Welfare benefits income ($100s)	.93 (.86 - 1.02)	-1.58	.11
Employment income ($100s)	1.00 (.93 - 1.07)	-0.02	.98
Told have HIV/AIDS by doctor	.28 (.14 - 0.59)	-3.38	.001
Housing status			.002
Live with sexual partner or friend [ref. Live on street, in car, single room]	2.72 (1.31 - 5.64)	2.68	.007
Live with family or in own house/apartment [ref. Live on street, in car, single room]	1.58 (.82 - 3.06)	1.38	.17

^a ^All predictors achieved a p-value < .10 in univariate regression models for one or more of the outcomes. Number of cases with complete data used in multivariate analysis equals 391.

^b ^An odds ratio greater than 1 indicates that the event is more likely to occur among those having the predictor trait; an odds ratio less than 1 indicates that the event is less likely to occur among those having the predictor trait.

^c ^PROC Logistic (SAS Institute)--Logistic regression model--was used to fit multiple regression model to data.

## Discussion

Many researchers have suggested that structural factors such as federal housing policies that limit drug users' access to housing subsidies and the criminalization of drug use explain the over-representation of drug users among the homeless compared to other low-income inner-city residents [52,53]. Other researchers explain the difference in rates of homelessness among drug users and non-drug users as a result of drug users' addictions [54-57]. In other words, drug users may be more likely to be homeless because they have a harder time keeping a job due to absenteeism or poor performance, or because they become evicted for not paying rent or using drugs in their apartments. Little research has empirically explored the impact of policy and structural factors, however, because of the difficulty in measuring these in individual survey research. This study explores the impact of structural factors on drug using and non-drug using residents' housing status by looking at their access to housing subsidies and supportive housing programs.

In our study, having HIV/AIDS, being female, and being African American predicted greater access to housing subsidies. Surprisingly, having a criminal conviction did not negatively impact access to housing subsidies, perhaps because nearly 65% of the total sample had a criminal history. The extraordinarily high rate of criminal convictions among drug users and non-drug users in our sample reflects structural conditions in the U.S. in which poor, ethnic minority, inner-city residents are profoundly over-represented in the criminal justice system.

Contrary to our expectations, recent drug use also did not significantly affect access to housing subsides. While federal housing policies allow local housing authorities to consider drug convictions in decisions to deny access to or terminate receipt of federal housing subsidies, many states choose to opt out of this policy. Connecticut does not exclude those with drug convictions from receiving federal housing subsidies. This may explain the lack of association between drug use and access to housing subsidies. However, these results also suggest that contrary to some researchers' hypotheses [57], drug users are not less likely to seek services because their drug addictions take precedence over other activities. Recent drug users were just as likely to apply for federal housing subsidies as other low-income residents.

As expected, being HIV positive or mentally ill predicted access to supportive housing. Drug use, however, did not. This is surprising given that supportive housing programs are designed to meet the needs of chronically homeless including those who are substance users as well as those living with HIV or mental illness. Our qualitative research with supportive housing providers in Hartford confirms that many supportive housing programs are designated specifically for HIV positive persons [58]. Other programs are designed for those with serious mental illness or substance abuse. Given the scarcity of programs relative to need, it may be that supportive housing providers prioritize giving services to those who are mentally ill or dually diagnosed with mental illness and substance abuse disorders, whom they may consider more in need of or more deserving of limited supportive housing units than chronically homeless substance abusers without mental illness. Alternatively, there are additional funding sources for housing people living with HIV/AIDS (PLWHA) such as HOPWA and Ryan White [43]. Thus, the priority given those with HIV/AIDS may be due to the more ready availability of funding for such programs, which are restricted to PLWHA.

While not being barred access to subsidized or supportive housing, recent drug use was negatively associated with being housed, including living in own apartment or with family members, or living in supportive housing. Housing subsidies and scattered site supportive housing require drug users to find free market rental housing. Drug users may be less likely to pass criminal background and credit checks than non-drug users [24,25]. In other words, while there seems to be no structural gaps in terms of access to housing subsidies among drug users, landlords may be unwilling to rent to drug users because of poor employment histories, bad credit, or criminal convictions.

Results from this study suggest that more nuanced measures of housing lead to a greater understanding of the relationship between housing status and sexual risk. In particular, our more nuanced and empirically-derived housing measure suggests that those who are unstably housed because they live with acquaintances or sex partners have higher rates of sexual risk than those who are homeless. Our previously published qualitative results suggest that those who double up with friends, acquaintances or sex partners may be expected to contribute toward household expenses or share drugs in order to stay, and thus may turn to sex exchanges [21,59]. People doubled up with sex partners, friends or acquaintances constitute the "hidden homeless" and, according to our results, are in desperate need for HIV prevention services.

Our results also suggest that living in an acquaintance's or sex partner's apartment should be differentiated from those living in family members' apartments. Those who live with family members have risk profiles more similar to those who are living in their own home than those who live with acquaintances or friends. This finding is consistent with our previous qualitative research in which we found that drug users who lived with family members often reduced their drug use and sexual risk out of respect for non-drug using family members [59]. Similarly, a study comparing out of treatment crack using women found higher levels of crack use among those who were separated from their children, compared with those who cared for them at home [60].

Our study also lends support to the argument that stable housing decreases sexual risk. Risk levels among those who lived in their own apartments or with family members and those who live in supportive housing had similarly low rates of sexual risk behavior. However, as seen in our results, those who receive supportive housing differ in many respects from those who do not in that they are more likely to be mentally ill and live with HIV. Supportive housing, in fact, may be significantly reducing HIV risk among a challenging and high-risk population. It is also possible that HIV status may explain the lower sexual risk behaviors, as people living with HIV often decrease their sexual risk after diagnosis [61]. However, the significant positive relationship between HIV status and unprotected sex in our results does not support this hypothesis. Alternatively, supportive services help residents in these programs maintain their housing and thus, in the long run, maintain lower sexual risk behaviors. Longitudinal analyses of housing stability are needed to answer these questions.

The research presented here has several limitations that should be noted. First, results presented here are from cross-sectional data and, thus, causality cannot be inferred. We are currently analyzing longitudinal data that will provide more direct causal evidence regarding the impact of housing status on HIV risk. Second, in spite of our attempts to target sampling to accurately represent low-income residents in the two cities, there may be selection biases that differentiate our sample from the general population of low-income residents. In particular, our participants, given that they were available for day-time interviews, may have been more impoverished and had higher rates of incarceration and lifetime drug use than the general population of low-income residents. These differences may have masked significant effects between drug use and incarceration and access to housing subsidies or supportive housing.

It should also be noted that findings from this study are limited to the state of Connecticut and cannot be generalized to other states in the U.S. or other countries. As mentioned, state and local Public Housing Authorities have considerable discretion in terms of whether or not to exclude persons with drug convictions from receiving federal housing subsidies. Other states in which drug offenders are banned from receiving housing subsidies may show stronger associations between drug use, criminal convictions and access to housing subsidies. In addition, Connecticut has been relatively active in promoting supportive housing to the chronically homeless in comparison to other states. However, like many states, the number of new supportive housing units planned has not materialized due to budget shortfalls [62]. Budget shortfalls have been even more severe in other states, and it is necessary to examine how both funding priorities and budgetary constraints affect drug using and non-drug using low-income residents' access to supportive and subsidized housing in other locations. Finally, other countries may differ in the over-all affordability of housing and amount of public investment in housing, policies and laws regarding drug use, and access to mental health and substance use treatment that could affect the relationships between drug use, criminal convictions, mental illness, HIV, housing access and status, and HIV risk shown in our results.

## Conclusions

Results form this study suggest that providing low-income and supportive housing may be an effective structural HIV prevention intervention, particularly for drug users. However, our study also suggests that access to supportive housing and housing subsidies is limited, and that some low-income residents have more access to these programs than others. Structural interventions are needed to increase the availability and accessibility of low income housing for those least able to compete in the housing and job market. These include increasing the number of federal housing subsidies and affordable housing programs, as well as increasing the availability of low-threshold supportive housing that does not require abstinence from drugs or engagement in mental health services as a precondition to housing. Such interventions could have a significant effect on reducing disparities in HIV prevalence among poor, inner-city and ethnic minority populations.

## Competing interests

The authors declare that they have no competing interests.

## Authors' contributions

JD-G conceived of the study and participated in its design, coordination and analysis. TM conducted the statistical analyses. MC coordinated the study, helped manage data and helped in analysis and interpretation. MW helped in the study design and analysis. JO coordinated the qualitative phase of the project and helped interpret results. All authors read and approved the final manuscript.
